# The SQSTM1/*p62* of Pacific White Shrimp (*Litopenaeus vannamei*) Is Involved in the Oxidative Stress Induced by Ammonia Exposure

**DOI:** 10.3390/ani16111718

**Published:** 2026-06-04

**Authors:** Wei Lu, Junliang Luo, Leyuan Feng, Shuanghu Cai, Jichang Jian, Shiping Yang

**Affiliations:** Guangdong Provincial Key Laboratory of Aquatic Animal Disease Control and Healthy Culture, Fisheries College, Guangdong Ocean University, Zhanjiang 524088, China; 19875928797@163.com (W.L.); luojunliang1022@163.com (J.L.); 18888576737@163.com (L.F.); caish@gdou.edu.cn (S.C.); jianjc@gdou.edu.cn (J.J.)

**Keywords:** autophagy, apoptosis, aquaculture, environmental toxicant, histopathology

## Abstract

Ammonia exposure can induce oxidative stress in aquatic animals. The p62 protein is a selective autophagy receptor that participates in protein degradation and oxidative stress regulation. In this study, the role of *Lv-p62* in the response of *Litopenaeus vannamei* to ammonia exposure was investigated using RNA interference. The results showed that *Lv-p62* expression was significantly induced in the hepatopancreas, gills, and intestine of *L. vannamei* after ammonia exposure (*p* < 0.05). *Lv-p62* expression peaked at 6 h in the gills and 24 h in the intestine, whereas a biphasic response was observed in the hepatopancreas, with an initial peak at 12 h and a higher second peak at 48 h. In the RNAi experiment, *Lv-p62* knockdown altered the expression of antioxidant-related genes (*Trx*, *Gst*, and *Gpx*) in a tissue-specific manner, with *Gpx* expression being prominently increased in the gills and intestine but not in the hepatopancreas under ammonia exposure. Autophagy-related genes (*ATG4* and *ATG10*) also showed time-dependent and tissue-specific expression changes after *Lv-p62* knockdown. The expression of apoptosis-related genes, including *caspase 3* and *p53*, was tissue-specific and was generally lower in the dsRNA-*Lv-p62*+NH_3_ group than in the dsRNA-EGFP+NH_3_ group at most time points. Histopathological observations showed that hepatopancreatic acinar vacuolation and structural damage were alleviated, and the hepatopancreatic apoptosis rate was reduced in *L. vannamei* in the dsRNA-*Lv-p62*+NH_3_ group. These findings suggest that *Lv-p62* participates in the response of *L. vannamei* to ammonia exposure, possibly by regulating antioxidant defense, autophagy-related processes, and apoptosis, thereby affecting hepatopancreatic oxidative damage and tissue injury.

## Simple Summary

Ammonia accumulation is a common problem in shrimp aquaculture and can cause oxidative stress and tissue damage. p62 is a selective autophagy receptor involved in protein degradation and oxidative stress regulation, but its role in Pacific white shrimp (*Litopenaeus vannamei*) under ammonia exposure remains unclear. In this study, *Lv-p62* responded to ammonia exposure in different tissues. Knockdown of *Lv-p62* changed the expression of genes related to antioxidant defense, autophagy, and apoptosis, and reduced hepatopancreatic injury and apoptosis. These findings suggest that *Lv-p62* is involved in shrimp responses to ammonia stress and may help improve our understanding of how shrimp cope with ammonia stress in aquaculture.

## 1. Introduction

*Litopenaeus vannamei* has become one of the most economically valuable aquaculture species worldwide, but it is susceptible to multiple environmental stressors, including salinity, temperature and ammonia [1,2]. Notably, ammonia exposure is a major environmental stressor that severely impacts the health status of aquatic animals, thereby increasing susceptibility to pathogens and raising mortality rates [3]. In aquatic environments, ammonia occurs in two main forms, unionized NH_3_ and ionized NH_4_^+^, with their ratio largely regulated by water pH [4]. In this study, ammonium chloride (NH_4_Cl) was used to establish ammonia stress. At an experimental pH of around 8.0, dissolved NH_4_Cl dissociates and reaches chemical equilibrium between NH_4_^+^ and toxic free NH_3_ [4]. Since NH_3_ easily penetrates biological membranes, it is regarded as the primary toxic form responsible for impairing aquatic animal health [5]. Specifically, ammonia could lead to oxidative stress in *L. vannamei* by increasing the concentration of reactive oxygen species (ROS) in hemocytes, and subsequently induce hepatopancreas damage [6,7]. Excessive ROS accumulation induced by ammonia stress disrupts intracellular redox homeostasis, thereby causing oxidative damage [6,7]. Oxidative stress not only impairs growth performance and immune function of shrimp [8], but also leads to mass mortality resulting in substantial economic losses to the aquaculture industry. Therefore, it is of great practical significance to deeply explore the regulatory mechanisms of the antioxidant system in *L. vannamei* under ammonia stress.

Autophagy is the process by which cells degrade damaged or redundant cellular components through lysosomes and is essential for maintaining cellular homeostasis [9]. Sequestosome 1 (SQSTM1), also known as p62, is a selective autophagy cargo receptor that recognizes ubiquitinated proteins or damaged organelles as autophagic cargo and links them to autophagosomes for subsequent lysosomal degradation [10]. Notably, it also regulates the antioxidant response via the nuclear factor E2-related factor 2/antioxidant response elements (Nrf2/ARE) pathway [11]. Mechanistically, accumulated p62 can interact with Keap1, reduce Keap1-mediated repression of Nrf2, and promote Nrf2-dependent transcription of antioxidant genes. In addition, p62 is closely related to pro-inflammatory and anti-inflammatory pathways [12]. Previous studies have shown that *Lv-p62* possesses conserved PB1 and UBA domains, which are associated with p62 oligomerization and recognition of ubiquitinated cargo, respectively [13]. This structural conservation supports the potential functional similarity of *Lv-p62* to p62 homologs in other species. In addition to its role in autophagy, p62 has also been implicated in the regulation of antioxidant responses in several aquatic species, although the detailed mechanism in *L. vannamei* remains unclear. Given that oxidative stress is a key trigger of autophagy [14], *p62* may serve as a critical integrator linking autophagy and antioxidant responses under environmental stress.

Overexpression of p62 activated the Nrf2/ARE signaling pathway through the degradation of Keap1 [11]. In *Cristaria plicata*, RNA interference (RNAi) of p62 resulted in inhibiting the expression of *Nrf2* and NADH quinone oxidoreductase 1 (*NQO1*), indicating that *p62* promoted the Nrf2/ARE pathway to protect against oxidative stress [15]. Previous studies have shown that waterborne zinc exposure can alter the transcriptional responses of autophagy-related genes, including *p62*, in yellow catfish [16]. This may be associated with heavy metal-induced oxidative stress, which activates autophagy-related pathways and thereby modulates *p62* expression as part of the cellular clearance response. In common carp (*Cyprinus carpio*), two *p62* genes, *Ccp62-1* and *Ccp62-2*, were reported to enhance antibacterial and antiviral immune responses and improve host survival [17]. Although the function of *p62* had been extensively studied in a variety of organisms, the functions of *p62* in regulating of oxidative stress induced by ammonia in *L. vannamei* is still unclear.

In this study, RNAi-mediated knockdown was used to investigate the role of *Lv-p62* in *L. vannamei* under ammonia exposure. We hypothesized that *Lv-p62* participates in ammonia stress responses by modulating antioxidant defense, autophagy-related processes, and apoptosis. Antioxidant-, autophagy-, and apoptosis-related gene expression, hepatopancreatic tissue damage, and TUNEL-positive apoptotic signals were assessed to clarify the involvement of *Lv-p62* in ammonia stress responses. This study provides functional evidence for the role of *Lv-p62* in *L. vannamei* under ammonia exposure.

## 2. Materials and Methods

### 2.1. Experimental Animal

The *L. vannamei* (3.9 ± 0.2 g) used in this experiment were obtained from local farmers on Donghai Island, Zhanjiang City, Guangdong Province, China. Then, the shrimp were acclimated in three breeding buckets (1 m^3^) for a week with 150 shrimp maintained in each bucket during the acclimation period. The shrimp were reared under indoor conditions, fed twice daily, and only healthy individuals with normal swimming behavior, active feeding, intact appendages, and no visible signs of disease or injury were used for the experiment.

Formal experiments were carried out in 0.5 m^3^ breeding buckets filled with natural seawater that had been disinfected with Qianglvjing (trichloroisocyanuric acid; Wuhan Shuidingdang Technology, Wuhan, China) and fully aerated before use (total water volume: 0.3 m^3^). Water parameters were maintained appropriately: temperature at 26–28 °C, salinity at 28–30, and pH at approximately 8.0, and dissolved oxygen above 5.0 mg/L. Air pumps continuously aerated into the water to ensure sufficient dissolved oxygen. During the experiment, pH, temperature, salinity, dissolved oxygen, and ammonia concentration were monitored, and ammonia was maintained within the normal range except for the ammonia-exposure treatment. All animal procedures were approved by the Guangdong Ocean University Research Council (approval number: GDOU-LAE-2026-054; approval date: 5 December 2025).

### 2.2. Ammonia Exposure Experiment

Healthy shrimp were randomly selected for ammonia exposure treatment. A total of 180 healthy shrimp were prepared for this experiment. Among them, 90 shrimp were assigned to three tank replicates, with 30 shrimp per tank, while the remaining shrimp were used for 0 h baseline sampling before ammonia exposure and kept as backup individuals. Except for scheduled sampling, no shrimp were artificially replaced during the experiment. Ammonia exposure was applied using ammonium chloride (NH_4_Cl; Sangon Biotech, Shanghai, China) to achieve a final ammonia nitrogen concentration of 20 mg/L [18]. At 0 h before exposure and at 6, 12, 24, 48, and 72 h after ammonia exposure, 3 shrimp were randomly sampled from each replicate, and the hepatopancreases, gills, and intestines of *L. vannamei* were collected. These tissues were immediately stored in liquid nitrogen for subsequent total RNA extraction to determine the time-series expression of the *Lv-p62* gene.

### 2.3. Verification of Lv-p62 Knockdown Efficiency

The dsRNAs used in this study, including dsRNA-*Lv-p62* and dsRNA-EGFP, were synthesized using an in vitro transcription T7 kit for siRNA synthesis (TaKaRa, Dalian, China). The assays were performed in PCR tubes (Jet Biofil, Guangzhou, China), and the quality and concentration of dsRNAs were verified by 1% agarose gel electrophoresis and NanoDrop 2000 spectrophotometry (Thermo Fisher Scientific, Waltham, MA, USA) before injection. To verify the knockdown efficiency of *Lv-p62* in *L. vannamei* under non-ammonia conditions, shrimp were injected with dsRNA-*Lv-p62* only. A second injection of dsRNA-*Lv-p62* was administered 24 h after the first injection to maintain RNAi efficacy during the sampling period. At 0, 6, 12, 24, 48, and 72 h after the second injection, shrimp were randomly sampled, and the hepatopancreases, gills, and intestines were collected and immediately stored in liquid nitrogen. The knockdown efficiency of *Lv-p62* was subsequently evaluated by qRT-PCR as described in Section 2.5 and Section 2.6. 

### 2.4. Lv-p62 RNAi Experiment

Healthy shrimp were selected for the experiment as described above and divided into two groups, with three replicate culture tanks per group. A total of 180 healthy shrimp were used in the RNAi experiment, with 30 shrimp per tank. The first group was injected with 100 μL of 0.5 μg/g dsRNA-EGFP, and the second group was injected with 100 μL of 0.5 μg/g dsRNA-*Lv-p62*. Both groups received a second injection with the corresponding dsRNA at 24 h after the first injection to maintain RNAi efficacy during the subsequent ammonia exposure period, and ammonia exposure was initiated immediately after the second injection. The two groups were designated as the dsRNA-EGFP+NH_3_ group and the dsRNA-*Lv-p62*+NH_3_ group, respectively. At 0, 6, 12, 24, 48, and 72 h post ammonia exposure, three shrimp were randomly sampled from each replicate at each time point, and the hepatopancreases, gills, and intestines were dissected and collected. These tissues were immediately stored in liquid nitrogen for subsequent total RNA extraction. No shrimp were artificially removed or replaced during the experiment. During each sampling period, only healthy and active shrimp were randomly selected for tissue collection. The dsRNA injection dose and basic RNAi procedure used in this study were based on our previously published study [13], with minor modifications according to the present experimental design.

### 2.5. RNA Extraction

Total RNA was isolated from shrimp tissues using a commercial RNA extraction kit (TaKaRa, Dalian, China) according to the manufacturer’s protocol. Subsequently, first-strand cDNA was synthesized from the extracted RNA using a reverse transcription kit (TaKaRa, Dalian, China) following the supplied instructions.

### 2.6. Gene Expression Analysis

Using EF-1α as the reference gene, the expression of the *Lv-p62* gene in *L. vannamei* was detected in the ammonia exposure experiment. After injection with dsRNA-*Lv-p62* or dsRNA-EGFP followed by ammonia exposure, the expression levels of antioxidant, autophagy, and apoptosis genes in *L. vannamei* were detected. The primer sequences used for these genes were designed based on sequences validated in our previous study [13] and are listed in Table A1. The relative expression levels of genes were calculated using the 2^−ΔΔCt^ method. Amplification was performed in a 10 μL total reaction volume with the QuantStudio 6 Flex qRT-PCR system (Thermo Fisher Scientific, Waltham, MA, USA) and TB Green^®^ Premix Ex Taq™ II (Tli RNaseH Plus; TaKaRa, Dalian, China), following the respective manufacturers’ protocols.

### 2.7. Histopathological Analysis

In the *Lv-p62* RNAi experiment, hepatopancreas samples from the dsRNA-EGFP+NH_3_ and dsRNA-*Lv-p62*+NH_3_ groups were collected at 72 h. Samples were fixed in Carnoy’s fixative (prepared in-house) for over 24 h and then processed for routine paraffin embedding as previously described with minor modifications [13]. Briefly, tissues were dehydrated through a graded ethanol series (70%, 80%, 90%, 95%, and 100%), cleared in xylene, infiltrated with molten paraffin at 60 °C, embedded in paraffin blocks, sectioned at 8 μm, rehydrated, and stained with hematoxylin-eosin (H&E) using a commercial kit (G1125, Solarbio, Beijing, China). Representative images were selected from multiple randomly observed sections to describe the main histopathological alterations.

### 2.8. Detection of Apoptotic Cells by TUNEL Assay

After deparaffinization and rehydration, tissue sections without HE staining were subjected to TUNEL staining with Elabscience’s One-Step TUNEL In Situ Apoptosis Detection Kit (Green, FITC) (Elabscience, Wuhan, China), following the manufacturer’s protocol, and then observed and image acquisition were conducted using an instrument from Leica Microsystems (Leica, Wetzlar, Germany).

### 2.9. Data Visualization and Statistical Analysis

All data are expressed as mean ± standard deviation (SD). Statistical analyses were performed using GraphPad Prism version 9.5. For the ammonia exposure experiment, the relative expression of *Lv-p62* at different time points within the same tissue was compared using one-way analysis of variance (ANOVA) followed by Tukey’s honestly significant difference (HSD) post hoc test. Different letters above the bars indicate significant differences among different time points within the same tissue (*p* < 0.05).

For the *Lv-p62* RNAi experiment, comparisons between the dsRNA-EGFP+NH_3_ group and the dsRNA-*Lv-p62*+NH_3_ group at the same time point were performed using an unpaired two-tailed Student’s *t*-test. Asterisks indicate significant differences between the two groups at the same time point (* *p* < 0.05, ** *p* < 0.01). No dataset was analyzed using both one-way ANOVA and Student’s *t*-test. The final design and layout of all figures were completed using Adobe Photoshop 2021 version 22.5.9. The statistical analysis procedures used in this study were based on our previously published study [13], with minor modifications according to the present experimental design.

## 3. Results

### 3.1. Tissue Expression Sequence of Lv-p62 After Ammonia Exposure

**Figure 1:**
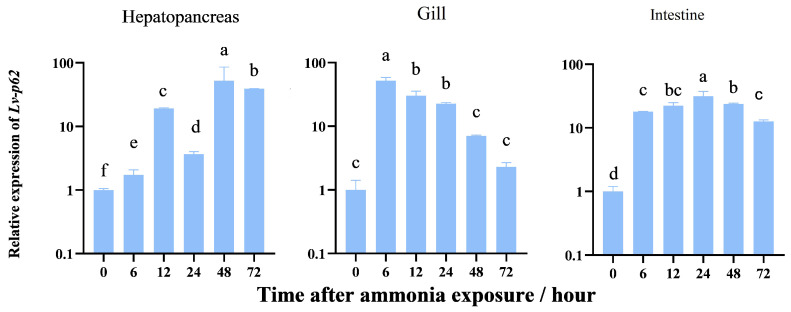
Tissue-expression dynamics of the *Lv-p62* gene in Pacific white shrimp (*Litopenaeus vannamei*) under ammonia exposure. Relative *Lv-p62* expression levels were detected in the hepatopancreas, gill, and intestine at different time points after ammonia exposure. Different letters indicate significant differences among time points (*p* < 0.05).

As shown in Figure 1, the mRNA expression level of *Lv-p62* in the hepatopancreas, gills, and intestine of *L. vannamei* was increased after ammonia exposure (*p* < 0.05). In the hepatopancreas, *Lv-p62* expression first increased to reach the first peak at 12 h, then decreased at 24 h, and subsequently rose again to reach the second, higher peak at 48 h, followed by a slight decline at 72 h. In the gills, *Lv-p62* expression peaked at 6 h (*p* < 0.05), followed by a gradual decrease in expression, and the level at 72 h remained higher than that at 0 h. In the intestine, *Lv-p62* expression increased continuously and peaked at 24 h, then declined gradually. These results indicate that ammonia exposure increased *Lv-p62* expression in a tissue-specific and time-dependent manner.

### 3.2. RNAi Efficiency Analysis

**Figure 2:**
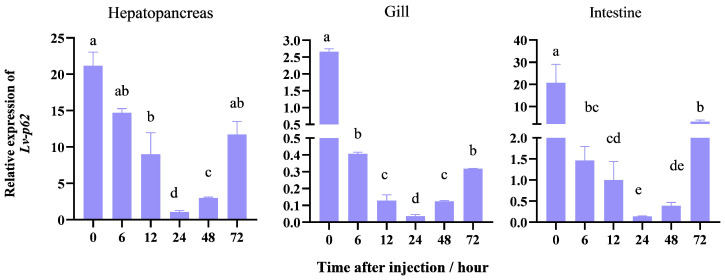
RNA interference efficiency analysis of *Lv-p62* in Pacific white shrimp (*Litopenaeus vannamei*). Relative *Lv-p62* expression levels were detected in the hepatopancreas, gill, and intestine at different time points after double-stranded RNA injection. Different letters indicate significant differences among time points (*p* < 0.05).

The knockdown effect of *Lv-p62* was assessed by qRT-PCR analysis in the hepatopancreas, gills and intestine. The relative expression level of *Lv-p62* was reduced after dsRNA-*Lv-p62* injection in all three tissues and reached its lowest level at 24 h after the second injection among the examined time points (Figure 2). These results suggest that the silencing effect of *Lv-p62* was most pronounced at 24 h after the second injection under the present experimental conditions. At later time points, *Lv-p62* expression showed a partial recovery.

### 3.3. Antioxidant-Related Genes Expression

**Figure 3:**
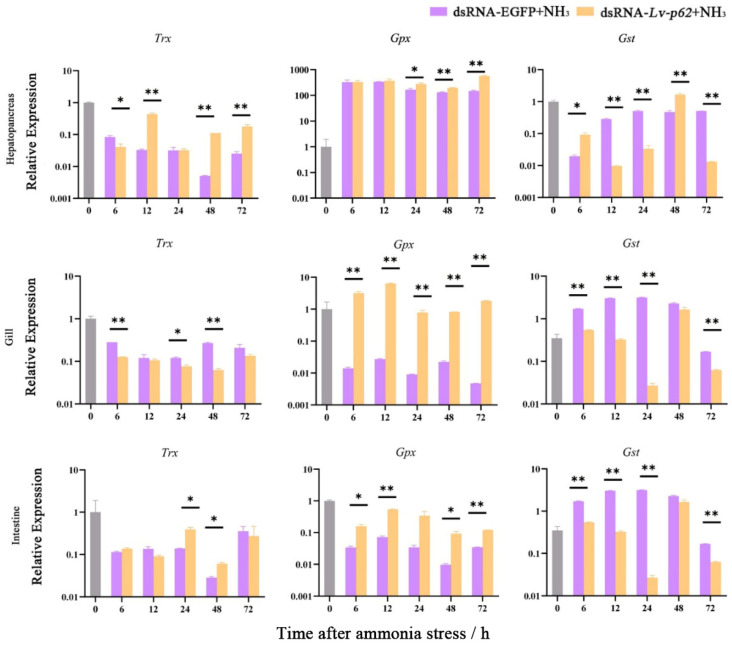
Expression levels of antioxidant-related genes in Pacific white shrimp (*Litopenaeus vannamei*) after ammonia stress. The relative expression levels of thioredoxin (*Trx*), glutathione peroxidase (*Gpx*), and glutathione S-transferase (*Gst*) in the hepatopancreas, gill, and intestine were detected by qRT-PCR at different time points after ammonia exposure. Asterisks indicate significant differences between the two treatment groups at the same time point, as determined by an unpaired two-tailed Student’s *t*-test (* *p* < 0.05, ** *p* < 0.01). Grey bars represent the control group before ammonia exposure (0 h).

As shown in Figure 3, antioxidant-related genes exhibited tissue-specific expression patterns after *Lv-p62* knockdown under ammonia exposure, with *Gpx* showing a prominent increase mainly in the gill and intestine. In the hepatopancreas, the *Trx* expression in the dsRNA-*Lv-p62*+NH_3_ group was significantly higher than that in the dsRNA-EGFP+NH_3_ group at 12 h, 48 h and 72 h (*p* < 0.05). *Gpx* expression in the dsRNA-EGFP+NH_3_ group decreased gradually to the lowest level at 48 h, whereas that in the dsRNA-*Lv-p62*+NH_3_ group peaked at 72 h (*p* < 0.05). *Gst* expression in the dsRNA-*Lv-p62*+NH_3_ group peaked at 48 h and then decreased, whereas that in the dsRNA-EGFP+NH_3_ group remained at a relatively high level after 24 h, with significant differences between the two groups at all detected time points (*p* < 0.05).

In the gills, *Trx* expression in the dsRNA-*Lv-p62*+NH_3_ group was significantly lower than that in the dsRNA-EGFP+NH_3_ group at 6, 24 and 48 h (*p* < 0.05), *Gst* expression in the dsRNA-*Lv-p62*+NH_3_ group was generally lower than that in the dsRNA-EGFP+NH_3_ group at all post-exposure time points from 6 to 72 h (*p* < 0.05), *Gpx* expression in the dsRNA-*Lv-p62*+NH_3_ group was significantly higher than that in the dsRNA-EGFP+NH_3_ group at all post-exposure time points from 6 to 72 h (*p* < 0.05).

In the intestine, *Trx* expression in the dsRNA-*Lv-p62*+NH_3_ group was significantly higher than that in the dsRNA-EGFP+NH_3_ group at 24 h and 48 h (*p* < 0.05), *Gpx* expression in the dsRNA-*Lv-p62*+NH_3_ group was significantly higher than that in the dsRNA-EGFP+NH_3_ group at 6, 12, 48 and 72 h (*p* < 0.05), and *Gst* expression in the dsRNA-*Lv-p62*+NH_3_ group was significantly lower than that in the dsRNA-EGFP+NH_3_ group across all time points (*p* < 0.05), with *Gst* expression in the dsRNA-*Lv-p62*+NH_3_ group reaching the minimum at 24 h while that in the dsRNA-EGFP+NH_3_ group peaked at 24 h.

### 3.4. Autophagy-Related Genes Expression

**Figure 4:**
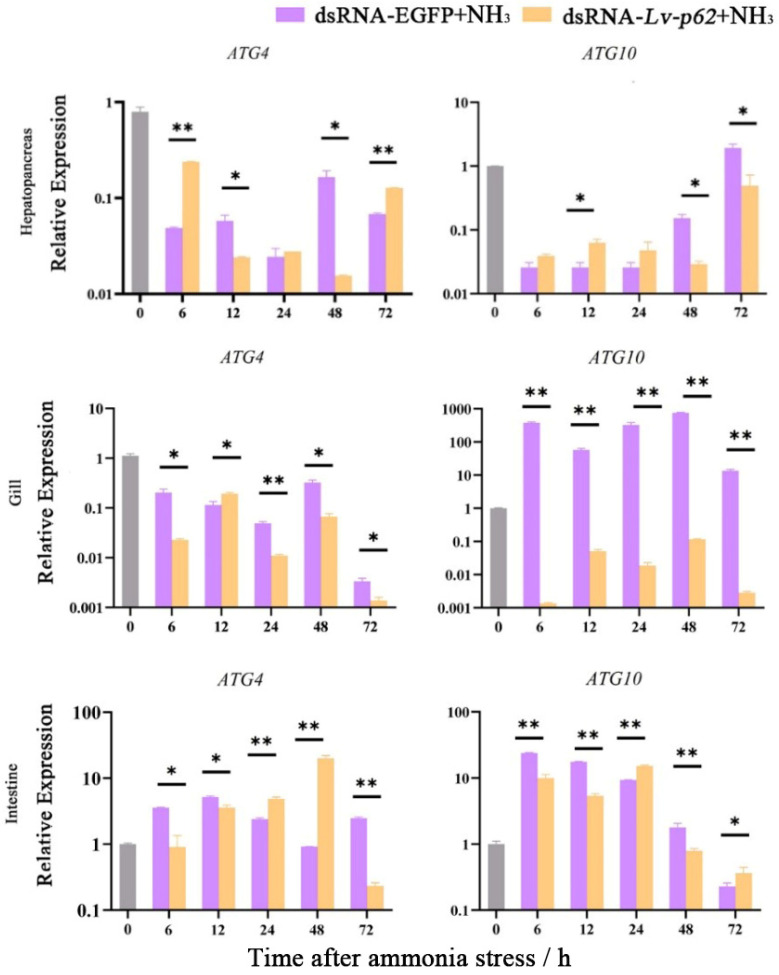
Expression levels of autophagy-related genes in Pacific white shrimp (*Litopenaeus vannamei*) after ammonia stress. The relative expression levels of autophagy-related gene 4 (*ATG4*) and autophagy-related gene 10 (*ATG10*) in the hepatopancreas, gill, and intestine were detected by qRT-PCR at different time points after ammonia exposure. Asterisks indicate significant differences between the two treatment groups at the same time point, as determined by an unpaired two-tailed Student’s *t*-test (* *p* < 0.05, ** *p* < 0.01). Grey bars represent the control group before ammonia exposure (0 h).

As exhibited in Figure 4, in the hepatopancreas, the expression of *ATG10* at all observation time points (6, 12, 24, 48 h) in the dsRNA-EGFP+NH_3_ group was lower than that at 0 h, and the *ATG10* expression levels in both the dsRNA-*Lv-p62*+NH_3_ group and the dsRNA-EGFP+NH_3_ group peaked at 72 h. Compared with the dsRNA-EGFP+NH_3_ group, the expression of *ATG10* in the dsRNA-*Lv-p62*+NH_3_ group was significantly downregulated at 48 h and 72 h. The *ATG4* expression in the dsRNA-EGFP+NH_3_ group decreased during the early stage after ammonia exposure, then increased and reached its highest level at 48 h. In contrast, *ATG4* expression in the dsRNA-*Lv-p62*+NH_3_ group was suppressed during the early stage and did not rebound until 72 h.

In gill tissue, the expression levels of *ATG10* and *ATG4* in the dsRNA-*Lv-p62*+NH_3_ group showed a downward trend after ammonia exposure, and were significantly lower than those in the dsRNA-EGFP+NH_3_ group (*p* < 0.05). The expression level of *ATG10* in the dsRNA-EGFP+NH_3_ group initially increased and remained significantly higher than that at 0 h until 72 h (*p* < 0.05).

In the intestine, *ATG10* and *ATG4* in both the dsRNA-EGFP+NH_3_ group and the dsRNA-*Lv-p62+*NH_3_ group first increased and then decreased after ammonia exposure. *ATG4* in the dsRNA-EGFP+NH_3_ group reached its highest level at 12 h, whereas that in the dsRNA-*Lv-p62*+NH_3_ group peaked at 48 h and then decreased.

### 3.5. Apoptosis Gene Expression

**Figure 5:**
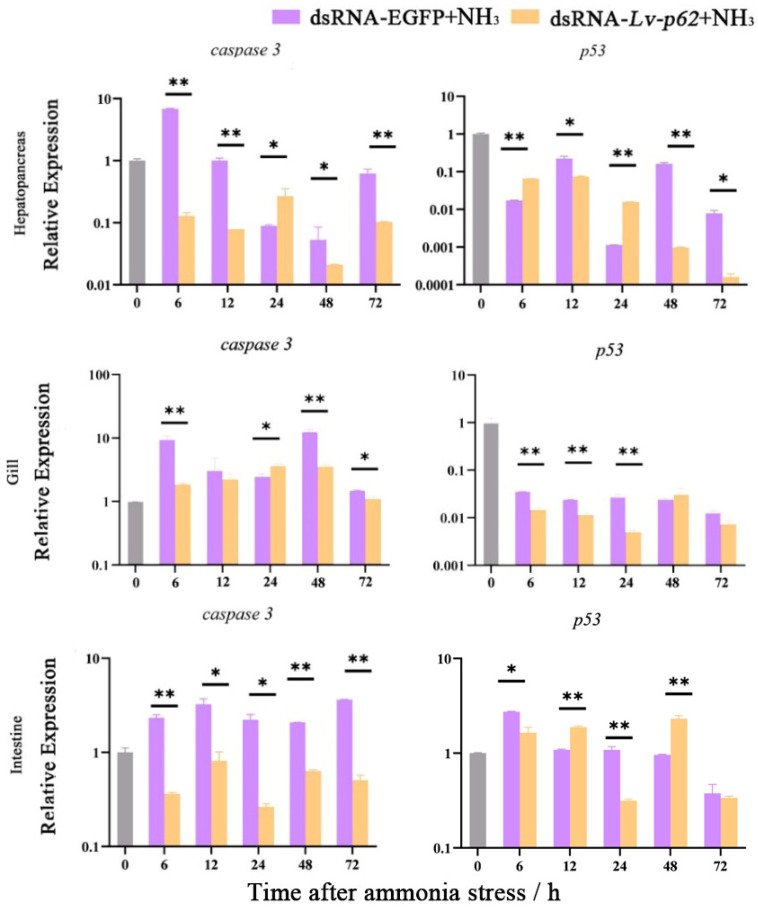
Expression levels of apoptosis-related genes in Pacific white shrimp (*Litopenaeus vannamei*) after ammonia stress. The relative expression levels of *caspase 3* and tumor protein *p53* in the hepatopancreas, gill, and intestine were detected by qRT-PCR at different time points after ammonia exposure. Asterisks indicate significant differences between the two treatment groups at the same time point, as determined by an unpaired two-tailed Student’s *t*-test (* *p* < 0.05, ** *p* < 0.01). Grey bars represent the control group before ammonia exposure (0 h).

As shown in Figure 5, in the hepatopancreas, for the *caspase 3* gene, the overall expression level in the dsRNA-EGFP+NH_3_ group was significantly higher than that in the dsRNA-*Lv-p62*+NH_3_ group at 6, 12, 48, and 72 h (*p* < 0.05). However, at 24 h, the opposite pattern was observed: *caspase 3* expression was significantly higher in the dsRNA-*Lv-p62*+NH_3_ group than in the dsRNA-EGFP+NH_3_ group (*p* < 0.05), and reached its peak in the dsRNA-*Lv-p62*+NH_3_ group. The *p53* expression in the dsRNA-*Lv-p62*+NH_3_ group was significantly lower than that in the dsRNA-EGFP+NH_3_ group at 12, 48, and 72 h (*p* < 0.05).

In the gill, the *caspase 3* in dsRNA-EGFP+NH_3_ group reached its peak expression at 48 h, while the dsRNA-*Lv-p62*+NH_3_ group peaked at 24 h and 48 h. Additionally, the *caspase 3* expression level in the dsRNA-*Lv-p62*+NH_3_ group was significantly lower than that in the dsRNA-EGFP+NH_3_ group at 6, 48, and 72 h (*p* < 0.05). *p53* expression levels in both the dsRNA-EGFP+NH_3_ and dsRNA-*Lv-p62*+NH_3_ groups were significantly lower than those at 0 h (*p* < 0.05).

In the intestine, the expression levels of *caspase 3* in the dsRNA-EGFP+NH_3_ were significantly upregulated after ammonia exposure (*p* < 0.05). The expression level of *caspase 3* in the dsRNA-EGFP+NH_3_ group was significantly higher than that in the dsRNA-*Lv-p62*+NH_3_ group at 6, 12, 24, 48, and 72 h (*p* < 0.05). For the *p53* gene, the dsRNA-EGFP+NH_3_ group reached its peak expression at 6 h, followed by an overall downward trend. In contrast, the dsRNA-*Lv-p62*+NH_3_ group showed fluctuating expression, with the highest level observed at 48 h.

### 3.6. Effects of Lv-p62 RNAi on Hepatopancreatic Histopathology

**Figure 6:**
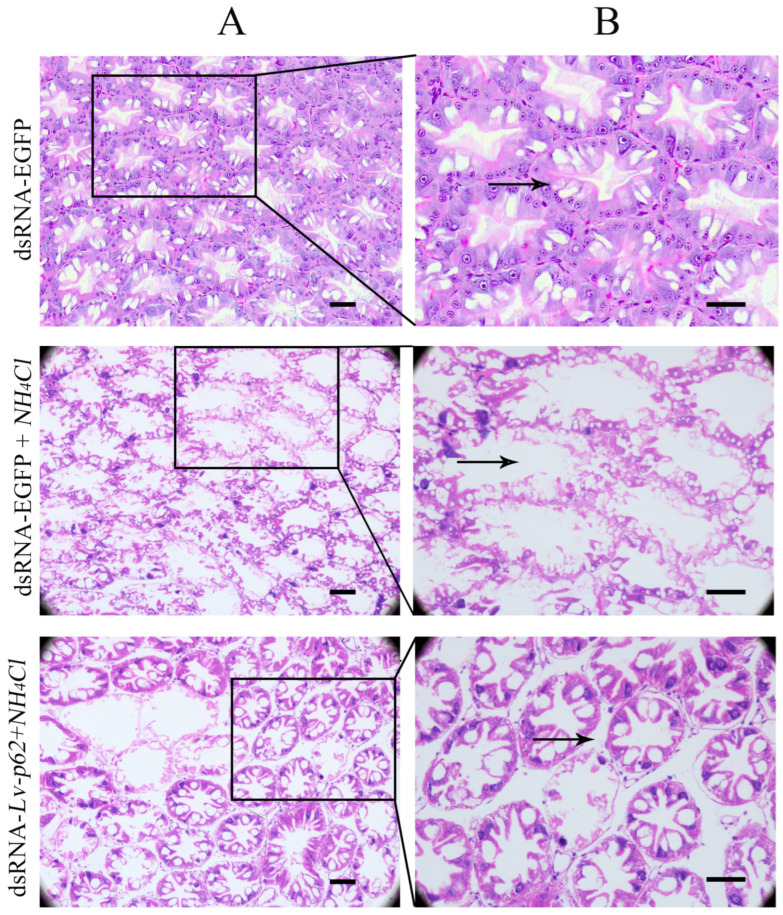
Histological changes in the hepatopancreas of Pacific white shrimp (*Litopenaeus vannamei*) among the three treatment groups. Notes Representative hematoxylin and eosin (H&E)-stained sections are shown for the dsRNA-EGFP group, the dsRNA-EGFP+NH_4_Cl group, and the dsRNA-*Lv-p62*+NH_4_Cl group. Panels (**A**) and (**B**) show the hepatopancreatic tissue at 200× and 400× magnification, respectively. Black boxes indicate the regions enlarged in the corresponding higher-magnification images. Arrows indicate hepatopancreatic tubule cavitation, lumen enlargement, and thinning of the tubule wall. Scale bars = 50 μm.

As observed in Figure 6, in the dsRNA-EGFP group, the hepatopancreatic acini were star-shaped with intact structures. However, in the dsRNA-EGFP+NH_3_ group, the walls of hepatopancreatic acini became thinner and distorted, and the normal structure was lost, with obvious vacuolation. The histopathological alterations were mainly characterized by acinar wall thinning, structural distortion, loss of normal architecture, and vacuolation, whereas obvious necrosis or epithelial sloughing was not observed in the examined sections. In contrast, vacuolation of hepatopancreatic acini in the dsRNA-*Lv-p62*+NH_3_ group was reduced, and the acinar structure was largely preserved, with morphology closer to that of the dsRNA-EGFP group.

### 3.7. TUNEL Detection Analysis

**Figure 7:**
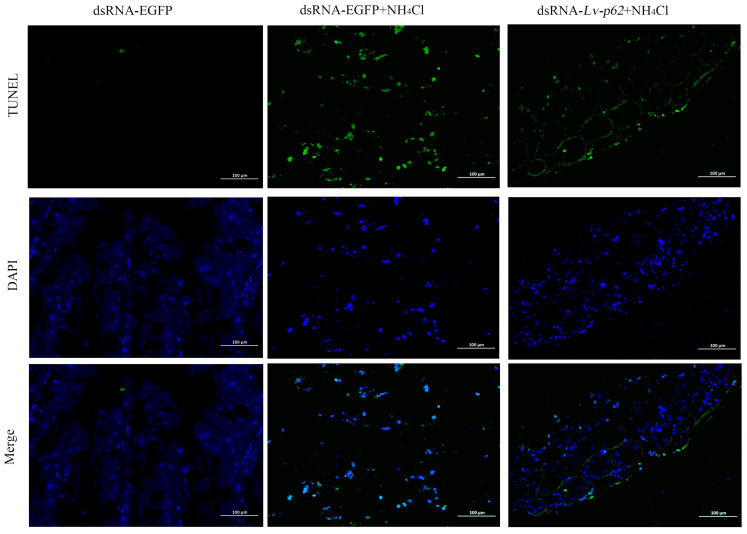
TUNEL staining of apoptotic hepatopancreatic cells in Pacific white shrimp (*Litopenaeus vannamei*) among the three treatment groups. Notes: Representative TUNEL-stained hepatopancreatic sections are shown for the dsRNA-EGFP control group, the dsRNA-EGFP+NH_4_Cl group, and the dsRNA-*Lv-p62*+NH_4_Cl group. Green fluorescence indicates TUNEL-positive apoptotic cells, blue fluorescence indicates DAPI-stained nuclei, and merged images show the colocalization of apoptotic signals and nuclei. All sections were observed at 200× magnification. Scale bars are shown in each panel.

**Figure 8:**
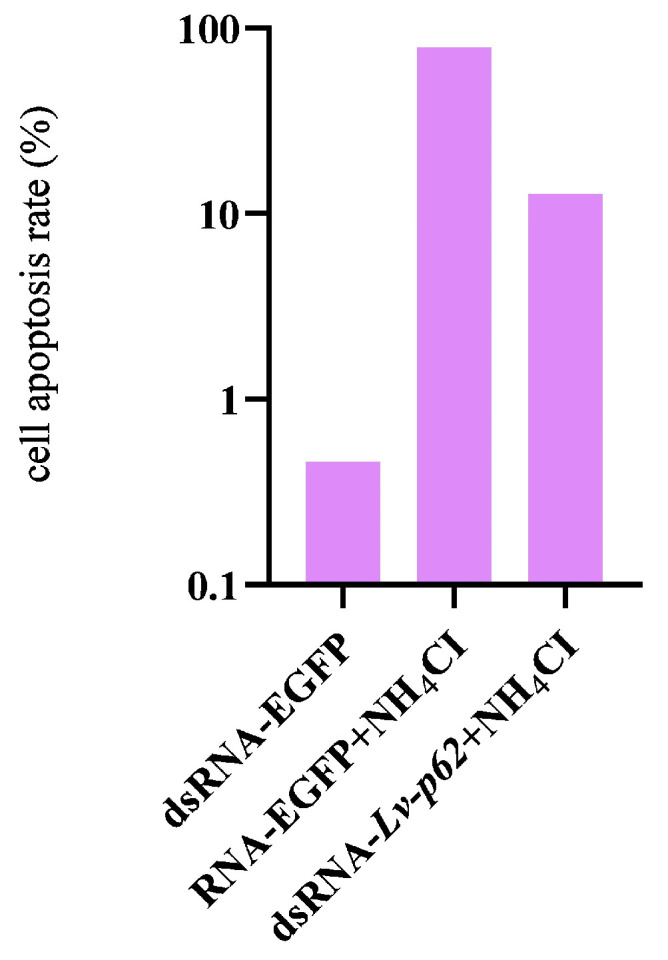
Quantitative analysis of hepatopancreatic cell apoptosis in Pacific white shrimp (*Litopenaeus vannamei*) based on TUNEL staining. The apoptosis rate was calculated as the percentage of TUNEL-positive cells relative to the total number of DAPI-stained nuclei in the analyzed fields. The three groups correspond to the dsRNA-EGFP group, the dsRNA-EGFP+NH_4_Cl group, and the dsRNA-*Lv-p62*+NH_4_Cl group.

As illustrated in Figure 7, apoptotic signals were detected by the TUNEL assay, with green fluorescence indicating apoptotic cells. Representative images from multiple randomly selected sections are shown. More obvious apoptotic signals were observed in the dsRNA-EGFP+NH_3_ group, whereas relatively weaker apoptotic signals were observed in the dsRNA-EGFP group and the dsRNA-*Lv-p62*+NH_3_ group. Quantitative analysis further confirmed that the cell apoptosis rate was increased in the dsRNA-EGFP+NH_3_ group, whereas this increase was reduced in the dsRNA-*Lv-p62*+NH_3_ group (Figure 8).

## 4. Discussion

P62 acts as a selective autophagy receptor and mediates protein aggregate degradation via its functional domains, thereby contributing to intracellular homeostasis [19,20]. p62 also activates the Nrf2 pathway to upregulate antioxidant gene expression. For example, exercise-induced upregulation of antioxidant proteins depends on p62-mediated Nrf2 activation [21]. In addition, p62-mediated selective autophagy contributes to immune regulation and host defense [22]. Our previous studies showed that *Lv-p62* was involved in the immune response against *Vibrio harveyi* infection [13]. However, the role of *Lv-p62* in the response of *L. vannamei* to ammonia exposure remains unclear.

Ammonia exposure causes tissue damage, oxidative stress, and metabolic disorders in *L. vannamei* [1]. We performed ammonia exposure and *Lv-p62* RNAi experiments, measuring gene expression in the hepatopancreas, gills, and intestine to evaluate the involvement of *Lv-p62* in ammonia-induced oxidative stress. After ammonia exposure, *Lv-p62* expression was significantly upregulated in all three tissues. Similar upregulation of p62 has been reported under starvation-induced oxidative stress in the intestine of *Pelodiscus sinensis* [23]. However, the peak expression time of *Lv-p62* differed markedly among tissues, peaking at 6 h in the gill, 24 h in the intestine, whereas a biphasic response was observed in the hepatopancreas, with a first peak at 12 h and a higher second peak at 48 h. The biphasic expression of *Lv-p62* in the hepatopancreas may reflect a stage-dependent response to ammonia stress. The first peak at 12 h may indicate an early transcriptional response to acute ammonia-induced cellular stress, whereas the higher peak at 48 h may be associated with accumulated cellular damage and enhanced *Lv-p62*-related autophagy/ubiquitin-associated stress signaling [10,19]. This interpretation is consistent with the lower hepatopancreatic vacuolation and weaker TUNEL-positive signals observed in the dsRNA-*Lv-p62*+NH_3_ group than in the dsRNA-EGFP+NH_3_ group, suggesting that sustained *Lv-p62* upregulation may be associated with the hepatopancreatic response under ammonia exposure. Since p62 can interact with Keap1 and promote Nrf2-mediated antioxidant responses, the second peak may indicate activation of a p62-Keap1-Nrf2-related adaptive response to prolonged oxidative stress [11,15]. The different *Gpx* responses after *Lv-p62* knockdown may be related to tissue-specific physiological functions. The gill and hepatopancreas show different toxicity responses under ammonia exposure [2], while the hepatopancreas has important roles in metabolism and immune regulation [24]. Therefore, *Lv-p62* knockdown may affect antioxidant gene regulation differently among tissues, possibly through p62-related antioxidant signaling such as the Keap1–Nrf2 pathway [25]. Physiologically, the gill is the tissue directly exposed to ammonia, which may explain its earliest response in *Lv-p62* expression under ammonia exposure [2]. Cong et al. also reported that the gill was the fastest-responding tissue in *Ruditapes philippinarum* under ammonia exposure [26]. These results suggest that *Lv-p62* may be involved in ammonia-induced oxidative stress.

Our results revealed tissue-specific expression patterns of antioxidant genes following ammonia exposure and *Lv-p62* knockdown. Notably, *Gpx* expression in the dsRNA-*Lv-p62*+NH_3_ group was significantly higher than that in the dsRNA-EGFP+NH_3_ group in both the gill and intestine, whereas *Trx* and *Gst* expression mostly showed the opposite pattern. These changes suggest that *Lv-p62* knockdown did not simply suppress the antioxidant system, but disturbed the balance of antioxidant gene regulation in a tissue-dependent manner. Mechanistically, this response may be closely associated with the p62–Keap1–Nrf2 signaling axis. Under oxidative stress, accumulated p62 can interact with Keap1, competitively inhibit Keap1-mediated repression of Nrf2, and thereby promote Nrf2 stabilization and transcriptional activation of antioxidant and detoxification genes [25,27]. Ammonia-induced ROS accumulation may disrupt redox homeostasis, promote oxidative damage, and induce increased apoptotic signals [7]. In addition, ammonia nitrogen stress can induce oxidative stress and autophagy-related responses in the shrimp hepatopancreas [14]. In the present study, hepatopancreatic structural damage, vacuolation, and increased TUNEL-positive signals were observed under short-term ammonia exposure, whereas the dsRNA-*Lv-p62*+NH_3_ group showed milder histological alterations and weaker TUNEL-positive signals than the dsRNA-EGFP+NH_3_ group. These results suggest that *Lv-p62* may participate in the acute hepatopancreatic response to ammonia stress; however, whether this effect persists under long-term ammonia exposure requires further investigation. The increased *Gpx* expression in the gill and intestine after *Lv-p62* knockdown may represent a compensatory response to enhanced oxidative pressure or activation of alternative antioxidant pathways. In contrast, the hepatopancreas did not show the same *Gpx* upregulation, which may be related to its central metabolic and detoxification functions and its greater susceptibility to ammonia-induced cellular damage. Under severe or prolonged stress, hepatopancreatic cells may have limited capacity to further activate *Gpx* transcription after *Lv-p62* knockdown. Another possible explanation is that antioxidant regulation in the hepatopancreas depends more strongly on the p62–Keap1–Nrf2 axis, whereas the gill and intestine may activate additional compensatory pathways because they are directly exposed to environmental ammonia or involved in barrier defense. Conversely, as reported in previous studies, *p62* knockdown can indirectly upregulate certain antioxidant genes by restoring *FOXO1/3* expression [28]. Thus, the increased *Gpx* expression observed in the gill and intestine after *Lv-p62* knockdown may represent a compensatory response to enhanced oxidative pressure or activation of alternative antioxidant pathways [28], whereas the decreased or less responsive expression of *Trx* and *Gst* may reflect impaired Nrf2/Keap1-mediated transcriptional regulation [25,27]. These findings imply that *Lv-p62* modulates antioxidant responses in a tissue-specific manner, probably via the Nrf2 pathway.

The expression of *ATG4* and *ATG10* in the hepatopancreas in the dsRNA-EGFP+NH_3_ group and the dsRNA-*Lv-p62*+NH_3_ group showed a downward trend after ammonia exposure. The result is consistent with a previous report [14]. Compared with the dsRNA-EGFP+NH_3_ group, *ATG10* expression in the gill and intestine was significantly downregulated in the dsRNA-*Lv-p62*+NH_3_ group. We have previously found that knocking down *p62* led to the downregulation of autophagy gene expression [13]. These results indicate that p62 is involved in regulating autophagy.

The expression of *caspase 3* in all tested tissues in the dsRNA-EGFP+NH_3_ group was upregulated at 6 h after ammonia exposure. Ammonia and *Vibrio* infection might trigger apoptosis [13,29]. Overall, the expression of *caspase 3* and *p53* was higher in the dsRNA-EGFP+NH_3_ group than in the dsRNA-*Lv-p62*+NH_3_ group. This is similar to the results in the *Vibrio* infection experiment [13]. TUNEL analysis showed that the dsRNA-*Lv-p62*+NH_3_ group had fewer TUNEL-positive signals than the dsRNA-EGFP+NH_3_ group. Knockdown of p62 could reduce the apoptosis of U87MG human glioma cells [30]. Collectively, these results suggest that *Lv-p62* may be involved in apoptosis-related responses during ammonia exposure in *L. vannamei*.

For crustaceans, the hepatopancreas is an important organ with multiple functions [24]. Therefore, histological analysis in this study was focused on the hepatopancreas because it is a major immune, metabolic, and detoxification organ in crustaceans and is highly sensitive to ammonia-induced oxidative damage [7,24,31]. Ding et al. found that black shrimp exposed to acute ammonia exposure exhibited hepatopancreatic oxidative damage, and this result is similar to that observed in the present study [31]. Hepatopancreatic acini in the dsRNA-*Lv-p62*+NH_3_ group also exhibited vacuolation, but the degree of vacuolar damage was lower than that in the dsRNA-EGFP+NH_3_ group. These findings suggest that *Lv-p62* may be associated with hepatopancreatic apoptosis-related responses and tissue damage under short-term ammonia exposure. Qian et al. also found that liver damage in mice with *p62* knockdown could be effectively repaired at 48 h after APAP-induced liver injury [32]. These observations are consistent with the possibility that *Lv-p62* is involved in hepatopancreatic apoptosis-related responses and tissue damage under ammonia exposure. In the present experimental design, the effects of *Lv-p62* knockdown were evaluated under ammonia exposure by comparing the dsRNA-EGFP+NH_3_ and dsRNA-*Lv-p62*+NH_3_ groups. Therefore, the interpretation of these results was limited to the comparison between these two groups under short-term ammonia exposure. In addition, the newly added RNAi efficiency data under non-ammonia conditions confirmed the effective knockdown of *Lv-p62*, supporting the reliability of the RNAi strategy used in this study. Accordingly, the conclusions were framed to describe the involvement of *Lv-p62* in responses to ammonia exposure, rather than its basal function under non-ammonia conditions. Further studies should validate the proposed mechanism at the protein and functional levels, including protein abundance, enzyme activity, Nrf2 activation, determination of un-ionized ammonia levels, and quantitative histological assessment across multiple time points.

## 5. Conclusions

In this study, ammonia exposure combined with RNAi was used to investigate the function of *Lv-p62* in the ammonia exposure response of *L. vannamei*. Knockdown of *Lv-p62* altered the tissue-specific expression patterns of antioxidant-, autophagy-, and apoptosis-related genes, and alleviated ammonia-induced hepatopancreatic injury and apoptosis. These findings suggest that *Lv-p62* participates in mediating ammonia-induced oxidative stress and tissue damage in *L. vannamei*. Overall, this study provides new insight into the role of *Lv-p62* in ammonia stress adaptation in shrimp.

## Data Availability

Upon a reasonable request, the corresponding author will provide the data supporting the results of this study.

## Abbreviations

The following abbreviations are used in this manuscript:

SQSTM1	Sequestosome 1
Lv-p62	*Litopenaeus vannamei* p62
RNAi	RNA interference
dsRNA	Double-stranded RNA
EGFP	Enhanced green fluorescent protein
qRT-PCR	Quantitative real-time polymerase chain reaction
ROS	Reactive oxygen species
Nrf2	Nuclear factor E2-related factor 2
ARE	Antioxidant response elements
Keap1	Kelch-like ECH-associated protein 1
Trx	Thioredoxin
Gst	Glutathione S-transferase
Gpx	Glutathione peroxidase
ATG	Autophagy-related gene
caspase 3	Cysteinyl aspartate specific proteinase 3
p53	Tumor protein 53
HE	Hematoxylin–eosin
TUNEL	Terminal deoxynucleotidyl transferase dUTP nick-end labeling
EF-1α	Elongation factor 1 alpha
NH_3_	Ammonia
NH_4_Cl	Ammonium chloride

## Appendix A

The primers used for qRT-PCR are listed in Table A1.

**Table A1:** Sequences of primers used in this study.

Gene Names	Sequence (5′–3′)
*dsp62-T7F*	TAATACGACTCACTATAGGGAGGATCTCTGTGCTGCCTGCGAGTTTA
*dsp62-T7R*	TAATACGACTCACTATAGGGAGTGTCCAAGGACATTCCTTGTCTTTG
*dsEGFP-T7F*	TAATACGACTCACTATAGGGAGGTGCCCATCCTGGTCGAGCT
*dsEGFP-T7R*	TAATACGACTCACTATAGGGAGTGCACGCTGCCGTCCTCGAT
*p62-F*	ATGTCAGACGACAGAAGCATGAGCG
*p62-R*	TTATTTGTTGACTGGCTGAAGAATG
*qTrx-F*	TTAACGAGGCTGGAAACA
*qTrx-R*	AACGACATCGCTCATAGA
*qGst-F*	AAGATAACGCAGAGCAAGG
*qGst-R*	TCGTAGGTGACGGTAAAGA
*qGpx-F*	AGGGACTTCCACCAGATG
*qGpx-R*	CAACAACTCCCCTTCGGTA
*qAtg4-F*	CTTTGTTGGTGATGAGGTCGTCTA
*qAtg4-R*	ACTTGTCTGTTTCCACTTCCGTTT
*qAtg10-F*	CAATCACTCGGGTAAACTTCT
*qAtg10-R*	GGATGCTCTTGTTGCGTCAGG
*qP53-F*	ATCCCGTGGGATTCTCTCCA
*qP53-R*	ATTTGTGACGACCTGCCCAT
*qCaspase3-F*	AACCAAGGCATCCCTGTCA
*qCaspase3-R*	GGGTTTATTCTGAAGTTGTGGG
*EF1α-F*	GTATTGGAACAGTGCCCGTG
*EF1* *α* *-R*	ACCAGGGACAGCCTCAGTAAG
